# A Role for Serotonin in Modulating Opposing Drive and Brake Circuits of Impulsivity

**DOI:** 10.3389/fnbeh.2022.791749

**Published:** 2022-02-17

**Authors:** Stephanie S. Desrochers, Mitchell G. Spring, Katherine M. Nautiyal

**Affiliations:** Department of Psychological and Brain Sciences, Dartmouth College, Hanover, NH, United States

**Keywords:** serotonin, impulsivity, reward, inhibition, learning

## Abstract

Impulsivity generally refers to a deficit in inhibition, with a focus on understanding the neural circuits which constitute the “brake” on actions and gratification. It is likely that increased impulsivity can arise not only from reduced inhibition, but also from a heightened or exaggerated excitatory “drive.” For example, an action which has more vigor, or is fueled by either increased incentive salience or a stronger action-outcome association, may be harder to inhibit. From this perspective, this review focuses on impulse control as a competition over behavioral output between an initially learned response-reward outcome association, and a subsequently acquired opposing inhibitory association. Our goal is to present a synthesis of research from humans and animal models that supports this dual-systems approach to understanding the behavioral and neural substrates that contribute to impulsivity, with a focus on the neuromodulatory role of serotonin. We review evidence for the role of serotonin signaling in mediating the balance of the “drive” and “brake” circuits. Additionally, we consider parallels of these competing instrumental systems in impulsivity within classical conditioning processes (e.g., extinction) in order to point us to potential behavioral and neural mechanisms that may modulate the competing instrumental associations. Finally, we consider how the balance of these competing associations might contribute to, or be extracted from, our experimental assessments of impulsivity. A careful understanding of the underlying behavioral and circuit level contributions to impulsivity is important for understanding the pathogenesis of increased impulsivity present in a number of psychiatric disorders. Pathological levels of impulsivity in such disorders are likely subserved by deficits in the balance of motivational and inhibitory processes.

## Introduction

Impulsivity is generally conceived of as a deficit in inhibitory control, resulting in unwanted actions. However, impulsive behavior has many diverse presentations and complex neurobiological underpinnings (Dalley and Robbins, 2017; Strickland and Johnson, 2021). Many lines of work have fractionated impulsivity into a number of different subtypes and components, with dissociable biological bases (Winstanley et al., 2004; Robbins et al., 2012; Bari and Robbins, 2013; MacKillop et al., 2016; Dalley and Robbins, 2017; Nautiyal et al., 2017; Bailey et al., 2021). Impulsive choice is described as risky decision making, including discounting of delayed rewards. Alternatively, impulsive action is characterized by acting prematurely and/or the decreased ability to stop or withhold responding. In this review, we focus on the action component of impulsivity, exploring the idea that impulse control can be broadly described as competing circuits. One “drive” circuit encodes an initially learned response-reward outcome association, and the second “brake” circuit subserves an opposing and subsequently learned inhibitory association. The sum of the outputs of these circuits shapes the action plan determining whether to go or inhibit going. In particular, we highlight the role of serotonin signaling in modulating these oppositional circuits in the control of impulsive action.

Dysfunction in different nodes of these “drive” and “brake” neural circuits could result in the heterogeneity of phenotypic presentations of impulsivity. Therefore, careful dissection of the underlying behavioral and circuit level contributions to impulsivity is important for understanding the pathogenesis of increased impulsivity. This idea is highlighted in the dual systems and imbalance models of adolescent impulsivity which consider disproportionate development and changes in communication for brain areas involved in reward/motivation and inhibitory control (Somerville et al., 2010; Steinberg, 2010; Casey et al., 2011; Ellingson et al., 2013). Considering imbalance models in the context of preclinical studies aimed at understanding adult impulsivity could help elucidate different entry points to dysfunctional circuits responsible for pathological impulsivity.

This dual-systems perspective is also relevant to clinical populations with disorders in which impulsivity is dysregulated. For example, attention deficit hyperactivity disorder (ADHD) is characterized by inhibitory deficits, including increased impulsive action (Schachar and Logan, 1990; Nigg, 2001; Wright et al., 2014; Grandjean et al., 2021). Impulsivity is also a key phenotype found in substance use disorder, in which both reward system and inhibitory dysfunctions are present (Jentsch et al., 2014; Weafer et al., 2014). From the perspective of reward sensitivity, genetic risk for alcoholism is associated with increased sensitivity to sweet substances (Kampov-Polevoy et al., 2001, 2003). Poor inhibitory control is associated with sensitivity to amphetamines (Weafer and De Wit, 2013; Weafer et al., 2017) and chronic cocaine use (Fillmore and Rush, 2002). Increased impulsive action likely reduces the ability to withhold actions to obtain or consume drugs, though it is difficult to parse out the cause versus effect, as is common generally when studying psychiatric disorders. However, it is clear that impulsivity is both a predisposing factor and a result of drug use. Several studies which supports a role for impulsivity as a causal factor shows that subjects with familial history of drug dependence have higher impulsivity across many domains, including impulsive action (Acheson et al., 2011; Ersche et al., 2012; Kumar et al., 2018). Additionally, increased impulsivity and altered reward sensitivity are also found in gambling disorder (Sztainert et al., 2013; Hodgins and Holub, 2015; Wardell et al., 2015; Jiménez-Murcia et al., 2017; Ioannidis et al., 2019; Mestre-Bach et al., 2020), which, as a behavioral addiction, is free from the confound of pharmacological effects on these phenotypes. Indeed, Brevers et al. (2012) found that the severity of problem gambling was predicted by performance on a stop-signal test of impulsive action.

**FIGURE 1:**
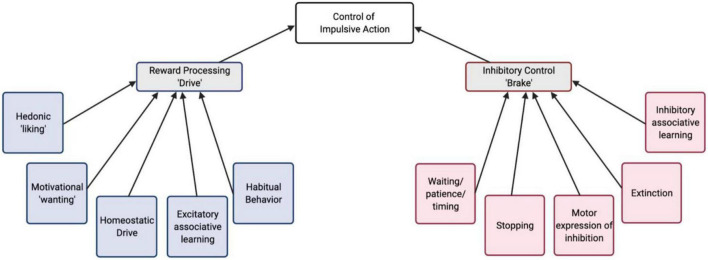
A conceptual schematic of behavioral/cognitive processes that contribute to the control of impulsive action. These are organized into reward “drive” and inhibitory “brake” processes.

Assuming the presence of competing drive and brake processes in impulsivity, we can examine the behavioral/cognitive components and the underlying neural mechanisms of each of these components. This sets up the possibility to arrive at the endpoint of increased impulsive behavior via a number of different paths and combinations of intermediate phenotypes (Figure 1). For example, in a behavioral assay of impulsive action, increased maladaptive actions could arise from a stronger action-outcome association, an increased motivation or valuation of reward, a failure to learn the opposing behavioral response (inhibition), or even a failure to express the inhibition, despite it having been learned. Understanding which components contribute to impulsive phenotypes, can lead toward developing novel, specific treatments targeting dysfunction of neural circuitry more precisely.

Serotonin (5-HT) has been strongly implicated in encoding reward *and* mediating behavioral inhibition, and is poised to modulate the balance of reward-based approach and adaptive inhibition of action. Manipulation of serotonin neuron activity in preclinical models clearly show that serotonin is involved in waiting and inhibiting behavioral responses (Wogar et al., 1992; Fletcher, 1995; Jolly et al., 1999; Winstanley et al., 2004; Fonseca et al., 2015; Miyazaki et al., 2018, 2020). Studies using *in vivo* monitoring, through single-unit electrophysiology and photometric calcium monitoring, in the dorsal raphe also implicate serotonin neurons in encoding both rewards and associated cues (Cohen et al., 2015; Li et al., 2016; Zhong et al., 2017; Ren et al., 2019). A large number of studies have also investigated the role of serotonin signaling—through many of its 14 receptors—in both reward-related behaviors and behavioral inhibition. Though many have used pharmacological approaches with systemically administered drugs, some studies have targeted brain region specificity with local drug administration and cell- and circuit-specificity using genetic models (Table 1). Given that serotonin, as a neuromodulator, tunes synaptic signaling and guides plasticity to alter learning and motivated behaviors, it is relevant to explore the idea that serotonin acts at the convergence of the neural circuits governing “drive” and “brake” processes in impulsivity.

**TABLE 1:** Effects of serotonin receptors on reward-related and impulsive action behavior from preclinical pharmacology and genetic mouse models.

Receptor	Behavioral effects	Reward	Behavioral inhibition: impulsive action
5-HT_1A_	Modulates anxiety, depression, and the antidepressant response to SSRIs	Activation enhances sensitivity to reward^1–3^	Agonists increase impulsive action likely through inhibition of raphe/serotonin signaling^4–6^
5-HT_1B_	Influences impulsive aggression and modulates social and drug reward	Activation reduces incentive motivation and knockout increases reward motivation^6–10^	Knockout increases impulsive action^8,11^
5-HT_2A_	Pro-hallucinogenic, necessary for psychedelic effects	Activation decreases incentive motivation^12,13^	Activation increases impulsive action, antagonists reduce impulsivity^14–17^
5-HT_2B_	Impulsivity, cognition, and anxiety	Knockout/blockade reduces reward sensitivity, and activation is required for some rewarding effects^18^	Knockout increases impulsivity^19^
5-HT_2C_	Influences feeding, stress, and sex behavior	Activation reduces incentive motivation^13^	Activation decreases impulsive action^15,17^
5-HT_3_	Nausea	Limited direct effects, but is necessary for the rewarding effects of MDMA, cocaine, morphine, and ethanol^12, 20–25^	No established effects
5-HT_4_	Anxiety, depression, and feeding	No effect^26^	No established effects
5-HT_5_	Memory and depression	No established effects	No established effects
5-HT_6_	Memory, activity, and anxiety	Limited direct effects^27^; striatal expression facilitates cocaine reinforcement^28–31^	No effect^16,32^
5-HT_7_	Depression and anxiety	No established effects	No established effects

*Blue and orange shading represent directionality (decreased or increased, respectively) of receptor activation effects on reward-related behaviors and impulsive action. ^1^Balleine et al. (1996), ^2^Fletcher et al. (1993), ^3^Fletcher et al. (1995), ^4^Groft et al. (2019), ^5^Miyazaki et al. (2012), ^6^Korte et al. (2017), ^7^Harrison et al. (1999b), ^8^Desrochers et al. (2021), ^9^Acosta et al. (2005), ^10^Fletcher et al. (2002), ^11^Pattij et al. (2003), ^12^Frick et al. (2015), ^13^Fletcher et al. (2017), ^14^Koskinen et al. (2000a), ^15^Silveira et al. (2020), ^16^Talpos et al. (2006), ^17^Fletcher et al. (2007), ^18^Doly et al. (2009), ^19^Goldman et al. (2010), ^20^Fletcher and Higgins (1997), ^21^Roger-Sánchez et al. (2013), ^22^Kelley and Hodge (2003), ^23^Rodd-Henricks et al. (2003), ^24^Rompré et al. (1995), ^25^Higgins et al. (1992), ^26^Reavill et al. (1998), ^27^Mitchell et al. (2007), ^28^Brodsky et al. (2016), ^29^Ferguson et al. (2008), ^30^Valentini et al. (2013), ^31^da Silva et al. (2018), and ^32^de Bruin et al. (2013).*

The first goal of this review is to synthesize studies, especially in preclinical animal models, that parse excitatory and inhibitory behavioral substrates that contribute to impulsive action. Next, we review potential circuit level mechanisms that underlie the interaction of these opposing learned associations in the generation of impulsivity. We focus on serotoninergic modulation of the underlying neural circuitry of both reward processing and inhibitory control, and also potentially in determining the balance of these competing systems to generate the output impulsive behavior. Finally, we discuss future research questions which examine the relative contributions of initial response-reward associations and subsequently learned inhibitory associations to increased impulsivity in terms of both behavioral substrates and the underlying neural circuits.

## The Drive: Contributions of Reward Processing to Impulsivity

Impulsivity is innately tied to reward processing, with excitatory drive being a key aspect of motivated behavior. Importantly, before being able to consider an inhibitory process, a motivated behavior needs to exist. This commonly includes a learned cue-reward or action-outcome association. In other words, we first learn to respond to obtain rewarding outcomes, prior to learning to avoid responding in certain circumstances (an innate or learned propensity to “go”). Alterations in appetitive associations may change the strength of the drive for reward. This could include differences in the intrinsic value/pleasurability of a reward (liking), and/or changes in the motivational value of the reward/reward paired cues (wanting). Though these are experimentally separable (Peciña, 2008; Berridge and Robinson, 2016; Morales and Berridge, 2020), they are linked together such that changes to either “liking” or “wanting” would likely increase actions in pursuit of reward, a characteristic of impulsive action. Therefore, superficially similar clinical presentations could actually be the result of dysfunction in different underlying neural mechanisms. Careful behavioral analysis using a variety of tests in different reward domains may allow us to identify the mechanisms contributing to pathological levels of impulsivity.

### Reward Processing in Classical Conditioning

Though impulsivity is defined in terms of operant behavior, in which impulsive behavior is characterized by actions that have unwanted consequences, the processes that underlie impulsive behavior may also be measurable at the level of Pavlovian tasks if they include changes to reward processing. In other words, if an instance of increased impulsivity was due to a change in the drive process, it may be able to be seen in altered appetitive classical conditioning, when outcomes are independent of action. For example, changes to the magnitude/value of an unconditioned stimulus influences the associative strength of conditioned cues, resulting in enhanced conditioned responding (Rescorla and Wagner, 1972; Pearce and Hall, 1980; Van Den Bos et al., 2004; Morris and Bouton, 2006). For appetitive conditioning, increased reward value due to altered hedonic pleasure or homeostatic processes could therefore increase the salience or associative strength of a cue, such that vigor of responding correlates with the perceived magnitude. If the value of a reward was subjectively increased, either due to pathological neural changes or simply everyday variations in reward preference in non-pathological cases, we would expect that subjects would form a stronger association between the cue and the reward and therefore have generally increased responding. For example, the phenomenon of signtracking, where animals may interact with a manipulable cue as if it were the reward which it has come to be associated with, shows that a classically conditioned cue can acquire increased incentive salience (Flagel et al., 2011). In fact, rats bred for a high novelty responding phenotype had increased signtracking behaviors along with a decreased ability to withhold responding in the differential reinforcement of low-rate responding test of impulsive action (Flagel et al., 2010). This interestingly correlates incentive salience with impulsivity – either subserved by a single underlying endophenotype or possibly due to a causal link of increased incentive salience leading to increased impulsive action. Interestingly high novelty responding rats also increased preference for the large reward in a delay discounting test of impulsive choice. Overall this study supports the idea that increased reward sensitivity may underlie both the operant impulsive and Pavlovian signtracking phenotypes. Additionally, in a study of excitatory Pavlovian responding during the adolescent developmental period, which is often characterized by heightened reward reactivity and impulsivity, adolescents showed increased responding under partial reinforcement conditions compared to adults (Meyer and Bucci, 2016a). This suggests that developmentally mediated impulsivity and altered classical conditioning may be modulated by similar reward-based changes. Taken together, the consideration of the processes which contribute to responding in appetitive classical conditioning may shed light on the mechanisms through which reward processing contributes to impulsive behavior.

Multiple neural substrates have been implicated in assigning value to an outcome or cue and incentive motivation. Dysregulation of any number of highly interconnected implicated brain regions could therefore result in altered reward related behavior. Several regions appear to represent or integrate reward value, including the nucleus accumbens (NAc), ventral pallidum (VP), basolateral amygdala (BLA), and regions of the prefrontal cortex including the orbitofrontal cortex (OFC) (Amiez et al., 2006; Chen et al., 2015; Howard et al., 2015; Wassum and Izquierdo, 2015; Ottenheimer et al., 2018). In particular, distinct areas of both the NAc (Peciña and Berridge, 2005; Peciña, 2008; Castro and Berridge, 2014) and the VP (Tindell et al., 2006; Ahrens et al., 2016; Richard et al., 2016; Smith et al., 2009) have been implicated in hedonic “liking” of reward assessed through taste reactivity, as well as incentive motivation “wanting.” The NAc is poised to integrate cortical and limbic information about reward and output to the VP, the subthalamic nucleus (STN), the substantia nigra, the ventral tegmental area (VTA), and the lateral hypothalamus, providing a mechanism for translating value assessment and motivation into behavior (Mogenson et al., 1983; Robbins and Everitt, 1996). Indeed, as reviewed by Day and Carelli (2007), the NAc and its connections are critical to appetitive Pavlovian cue-outcome learning, both in association acquisition and motoric expression. In sum, changes in brain regions involved in both “liking” and “wanting” aspects of reward processing could contribute to increased responding to conditioned stimuli during appetitive classical conditioning by subjectively increasing the outcome value.

#### Contributions of Serotonin to Classical Conditioning Through Modulation of Reward Processing

**FIGURE 2:**
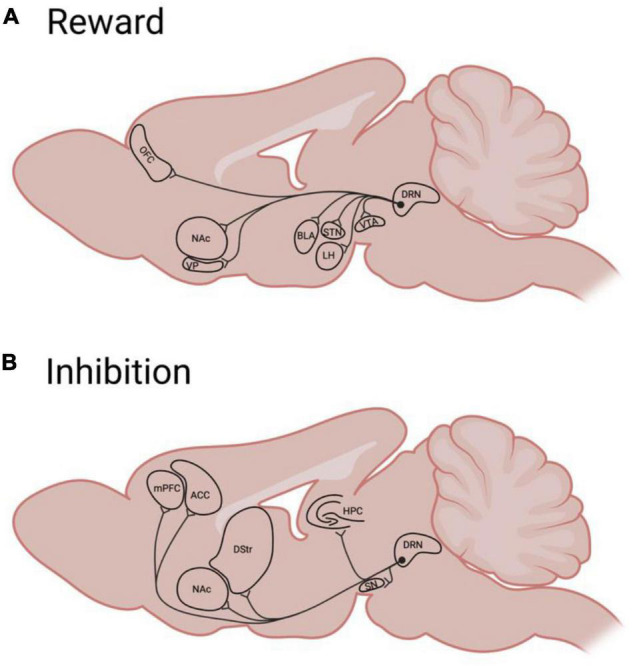
A simplified schematic of rodent Dorsal Raphé serotonergic efferents to brain regions implicated in reward **(A)** or behavioral inhibition **(B)**. ACC, anterior cingulate cortex; BLA, basolateral amygdala; DRN, dorsal raphé nucleus; DS, dorsal striatum; HPC, hippocampus; LH, lateral hypothalamus; mPFC, medial prefrontal cortex; NAc, nucleus accumbens; OFC, orbitofrontal cortex; STN, subthalamic nucleus; SN, substantia Nigra; VP, ventral pallidum; VTA, ventral tegmental area.

Many brain regions involved in reward encoding and classical conditioning are innervated by the serotonin system, rendering serotonin as a well-positioned modulator of reward processes (Huang et al., 2019; Ren et al., 2019; Figure 2A). Serotonergic neurons within the dorsal raphe nucleus (DRN) respond by increasing firing to both expected and unexpected rewards (Cohen et al., 2015; Li et al., 2016; Zhong et al., 2017), indicating that serotonin generally does not encode “surprise” or prediction-error. Rather, during classical conditioning, some DRN neurons develop a ramping response to reward-predictive cues, with response magnitude being commensurate to expected reward value (Nakamura et al., 2008; Bromberg-Martin et al., 2010). This response specifically requires that stimuli have acquired value through conditioning (Zhong et al., 2017), and differs from the response of other DRN neurons during aversive experiences (Hayashi et al., 2015). Thus, serotonergic signaling reflects the value (either learned, in the case of cues, or innate, in the case of rewards or punishments) of stimuli, with different populations (and projections; Ren et al., 2018) responding selectively to appetitive or aversive events.

Serotonin’s involvement in reward processes ultimately depends on not only the activity of serotonergic neurons, but also the projection targets as well as the receptors expressed in those areas. Brain regions within canonical reward circuitry—and containing “hedonic hotspots” (Peciña et al., 2006)—receive dense innervation from the DRN and express various serotonin receptors. Serotonin signaling in some of these reward processing regions, specifically the NAc and VP, appears to mediate the euphoric effects of recreational drugs (Yoshimoto et al., 1992, 2012; Napier and Istre, 2008; Matsui and Alvarez, 2018). However, the hedonic effects of serotonin signaling are not consistent across all receptors, and specifically targeting distinct receptors manifests varied, and sometimes opposite, effects (Table 1). Manipulating general serotonin tone via systemic agonism of 5-HT_1A_ receptors (thought to increase synaptic serotonin by antagonizing autoreceptors) or genetic knockout of serotonin transporters (SERT; also thought to generally increase synaptic serotonin, Homberg et al., 2007) fails to alter hedonic liking of palatable tastes (Treit and Berridge, 1990; Caras et al., 2008) or incentive wanting for reward paired cues (Nonkes et al., 2014). However, subjects with depleted serotonin levels display reduced neural responsivity to rewards in fMRI (Seymour et al., 2012), and we recently demonstrated that mice lacking the 5-HT_1B_ receptor seem to have increased subjective reward valuation, including enhanced hedonic taste reactivity to sucrose in a lickometer test (Desrochers et al., 2021). Taken in totality, this evidence suggests that serotonin likely does not have a unified brain-wide role in reward processing. Rather, to accurately characterize serotonin’s functions, region, cell-type, and receptor specificity must be considered.

Communication between subregions of the OFC and BLA is crucial in learning, representing, and using the value of cues to guide behavior (Malvaez et al., 2019; Lichtenberg et al., 2021; Sias et al., 2021), and serotonergic signaling regulates this communication. Serotonin neurons projecting to the BLA respond to both reward and punishment (Ren et al., 2018) and may have a general role in tuning the response of this region to stimuli; BLA serotonin activates GABAergic interneurons, which inhibit excitatory projection neurons (Rainnie, 1999; Bocchio et al., 2015), and should be expected to mute the area’s response to salient stimuli. Interestingly, the dampening effect on BLA activity appears to depend on balanced serotonin signaling; while acute administration of serotonin excites inhibitory interneurons, prolonged exposure to serotonin (such as would occur in the absence of proper serotonin re-uptake) *reduces* the inhibitory output of interneurons on BLA principal neurons (Rainnie, 1999). In agreement with this, reduced 5-HT levels following excitotoxic lesion or 5-HT desensitization, leads to amygdalar over-activity and over-responding to reward paired cues (Nonkes et al., 2010; Man et al., 2012). Serotonin in the OFC regulates anticipatory encoding of reward in response to predictive cues (Zhou et al., 2015) and coordinates emotional and behavioral responses to those cues (Man et al., 2012). Overall changes to serotonin signaling in these areas results in deficits in the ability to represent and use expected outcome values and increases the likelihood of an animal’s adopting inefficient behavioral strategies, such as seen in impulsivity.

Region-specific and receptor targeted parsing of the serotonin system is historically difficult, due to the system’s complexity and the limitations of some tools in distinguishing varied components. For example, serotonergic neurons can corelease glutamate throughout the brain (Sengupta et al., 2017; Ren et al., 2018; Belmer et al., 2019; Wang H. L. et al., 2019). Monitoring DRN activity doesn’t distinguish between the effect of serotonin and glutamate release, even when neurons are targeted in projection-specific approaches. Global manipulations of the serotonergic system, such as SERT KO or inhibition, produce manifold compensatory changes beyond simply altering the level of serotonin in the synapse (Homberg et al., 2007). Historically, the primary technique available for monitoring serotonin *in vivo* was microdialysis, which is sensitive to only one of the multiple timescales at which serotonergic neurons appear to operate (Cohen et al., 2015). Fortunately, recent advances in fluorescent-based in-vivo monitoring techniques now allow for direct monitoring of serotonin release at time scales compatible with understanding its role in reward processing, motivation, and impulsivity (Unger et al., 2020; Dong et al., 2021; Wan et al., 2021).

### Reward and Impulsive Action

In addition to classical conditioning, reward processing is central to instrumental behavior, and increased impulsivity could result from the overvaluation or increased motivation for reward, which override the negative consequences associated with taking action. Difficulty in withholding or stopping ongoing responding for reward in tests of instrumental behavior is a defining characteristic of impulsive action. Examples of paradigms used to assess this component of impulsivity include the Go/No-go (measuring the decreased ability to withhold responding when presented with a no-go cue), 5-choice serial reaction time test (5CSRTT; assessing premature responding), stop signal reaction time test (SSRT; testing the decreased ability to halt ongoing responding), and differential reinforcement of low rate responding (DRL; measuring the decreased ability to withhold responding for a wait period).

Importantly, an increased impulsive action phenotype may influence behavioral readouts in other operant paradigms testing motivation, such as random ratio and progressive ratio. Changes in excitatory responding (actions normally taken in pursuit of reward), for example the vigor of responding, which are subserved by changes in reward circuitry (Bailey et al., 2016, 2018) may make inhibiting the response more difficult. This could drive increased/disordered responding seen in these operant tasks, as well as in clinical cases of increased impulsivity. For example, dysfunctional reward processing is frequently comorbid in psychiatric disorders characterized by levels of increased impulsivity, including substance use, gambling disorders, and schizophrenia (Bjork et al., 2004; Monterosso et al., 2005; Rubio et al., 2008; Billieux et al., 2012). Preclinically, rats that show high levels of premature responding in the 5CSRTT are also more sensitive to cue-induced reinstatement of sucrose-seeking (Diergaarde et al., 2009). The question remains if the dysregulated impulsivity is causally linked to the reward system dysfunction. We recently developed a paradigm to show that increasing reward value on a trial-by-trial basis can lead directly to increased impulsive action in a Go/No-go paradigm (Desrochers et al., 2021). Future studies can expand on this to attempt to ameliorate disordered impulsivity by normalizing the aberrant reward processing.

Approaches to dissect the underlying neural circuits of co-occurring reward process and inhibitory dysfunction can determine if the neural circuit dysregulation is subserved by convergent mechanisms. Many of the same brain areas noted to be involved in reward and motivation have also been implicated in impulsive action. In particular, the NAc and its core and shell subregions have been extensively studied for their individual roles in impulsive action through reaction time tests, with pharmacological manipulations and deep brain stimulation of the shell subregion causing elevated premature responding (Sesia et al., 2010; Feja et al., 2014). Optogenetic stimulation of projections from the VTA to the NAc shell also increased premature responding during a long inter- trial interval in the 5CSRTT (Flores-Dourojeanni et al., 2021). Additionally, prefrontal cortical regions modulate impulsive action, though they are more often associated with assigning value to different decisions and choosing between actions (OFC, mPFC). Specifically, in an imaging study in humans, Mechelmans et al. (2017) found that impulsivity for high value reward cues in a 4CSRTT was accompanied by increased activity in the mOFC and in a monetary incentive delay task was associated with increased functional connectivity between the STN and left mOFC. In a rodent study of the 5CSRTT, rats that tended to respond prematurely had alterations in oscillatory patterns in the mPFC and NAc, which may cause abnormal reward encoding resulting in increased impulsive action (Donnelly et al., 2014).

The alterations in reward-related behavior in impulsivity could also be the result of impaired action selection supported by the dorsal striatum, which is important when there is an instrumental contingency between response and reward, as in many tests of impulsive action (Balleine et al., 2007; Corbit and Janak, 2007, 2010). Pharmacological manipulations of serotonin and glutamate receptors in the dorsal striatum modulate premature responding in the 5CSRTT (Agnoli and Carli, 2012). The varied regions associated with the control of impulsive action highlight the importance of considering reward processing in the study of impulsivity, as well as suggest that there may be many ways to cause an impulsive action “phenotype” through modulation of different behavioral endophenotypes. Behavioral analysis which considers the learning, hedonic, and motivational contributions to pathological cases of impulsivity may help clarify and point toward more specific neural targets for treatment.

#### Contributions of Serotonin to Impulsive Action Through Reward

Given that serotonin signaling is involved in many aspects of reward processing, it is relevant to consider how the influence of serotonin on reward contributes to its effect on impulsivity. As discussed in prior sections, most, if not all, rodent assays of action impulsivity involve approach toward appetitive cues and outcomes, and are therefore confounded by reward processing. As such, the effects of manipulations to serotonin signaling on tests of impulsive action may in some cases arise from effects on reward responsivity. Directly assessing the effects of experimental manipulations of serotonin signaling on reward related alterations is helpful to accurately interpret the effects in traditional tests of impulsivity.

Serotonin enables appropriate waiting for reward (Eagle et al., 2009; Miyazaki et al., 2014), and activation of serotonergic neurons in the DRN is correlated with (Miyazaki et al., 2011) and causally linked (Miyazaki et al., 2014) to waiting. The OFC, in addition to its previously described involvement in reward processing, mediates at least some of serotonin’s action in impulse control, as stimulating serotonin release in the OFC almost fully recapitulates the effect of DRN stimulation on waiting (Miyazaki et al., 2020). These two sets of functions are likely intertwined. Serotonergic signaling in the OFC is often associated with impulsive choice (Wischhof et al., 2011; Darna et al., 2015), in part because of its well established contributions to tracking and representing the value of reward predictive cues (Clarke et al., 2007; Nonkes et al., 2010). However, there is evidence that it is also involved in the capacity to withhold premature responses, to stop ongoing behavior, and to perform other forms of response inhibition (Chudasama et al., 2003; Eagle et al., 2008b; Mechelmans et al., 2017). Yet, the OFC is not necessary for pure motor inhibition (Swick et al., 2008) nor does it encode the value of actions themselves but rather that of affective stimuli (Rolls, 2004; Rudebeck et al., 2008). Thus, the understanding of OFC serotonergic function in impulsivity should not be restricted to a choice vs. action binary nor to a pure response inhibition framework. Rather, it appears related to the ability to withhold behavior generally and likely does so through its role in outcome value encoding.

Serotoninergic modulation of prospective reward encoding within the OFC is heterogeneous (Zhou et al., 2015), as are its effects on OFC neuronal activity (Wright et al., 2017, 2021). During reward prediction, OFC activity is distinct from baseline yet characterized by neither a gross increase nor decrease in activity (Shobe et al., 2017). This “activity-silent” state (Stokes, 2015) is similar to the heterogeneous responses observed in response to serotonin tone. The OFC communicates information regarding the prospective value of expected rewards to other limbic structures, its connectivity with the NAc being particularly important (Meyer and Bucci, 2016b; Wang Z. et al., 2019), thereby permitting the usage of that value in the computation of whether or not to initiate a behavioral response.

The mesolimbic pathway is another circuit in which serotonin regulates reward encoding and the generation of motivated behavior. In humans, genetic variation in a serotonin production enzyme, tryptophan hydroxylase-2, is associated with increased impulsivity and increased responsivity of NAc to reward (Neufang et al., 2016). Gross manipulations to serotonin tone within the limbic system in rodents alter motivational drive. For example, the impulsivity produced by serotonin depletion in the NAc does not appear to reflect an alteration in response inhibition (Fletcher et al., 2009). Specifically, while it increases the rate of responding in the DRL, it does not alter burst responding nor does it increase premature responding in the 5CSRTT. More specific manipulations to serotonergic signaling through either 5-HT_1B_ (by gene knockout) or 5-HT_2C_ receptors (by receptor antagonist) impairs response inhibition while also increasing mesolimbic DA release (Pennanen et al., 2013; Nautiyal et al., 2015), suggesting that these receptors may mediate the general effect of serotonin on NAc activity and behavior. The 5-HT_1B_ receptor is a likely substrate through which serotonin may influence reward and impulsive action: 5-HT_1B_ receptor knockout increases reward valuation in a lickometer test and false alarm rate for no-go trials in the Go/No-go test (Desrochers et al., 2021), and restoring 5-HT_1B_ receptor expression in adulthood reverses both action impulsivity and altered dopamine signaling in the NAc (Nautiyal et al., 2015). Interestingly, overexpression of 5-HT_1B_ receptors in NAc shell projections to the VTA also increases the rewarding effects of drugs of abuse (Neumaier et al., 2002; Furay et al., 2011). Additionally, reduced binding of these receptors in the NAc and VP is associated with major depressive disorder, of which insensitivity to reward (anhedonia) is a principal symptom (Murrough et al., 2011). More generally, 5-HT_1B_ signaling is linked to depression-like behavior in animal models (Svenningsson et al., 2006) and to cocaine or social reward through is localization in the NAc, (Dölen et al., 2013; Fontaine et al., 2021). Thus, 5-HT_1B_ under- *or* over-expression can contribute to altered reward processing, suggesting that normal function requires maintenance of balanced signaling.

In sum, there is evidence that serotonergic regulation of impulsivity occurs, in part, at the level of reward processing. Described above are proposed roles for serotonin in linking these processes in the OFC and mesolimbic pathway. However, the extent of serotonin’s involvement in such a link in many other regions remains to be characterized. Figure 2A summarizes areas targeted by serotonergic neurons that are involved in reward and impulsivity which may be promising targets for such characterization. Research that seeks to bridge the gap between reward and impulsivity—such as through the use of batteries of behavioral assays that provide information across multiple dimensions—would greatly enhance our understanding of not only impulse control, but also the processes by which motivated behaviors and impulses are generated.

## The Brake: Contributions of Inhibitory Control to Impulsivity

Alternatively to increased reward drive, disordered impulsivity can be considered as a failure of inhibitory processes, even colloquially described as a lack of “self-control.” In the impulsive action subtype of impulsive behavior, this presents as deficits in preventing responding or stopping ongoing responding. Withholding an action, or learning that the absence of response results in reward, is an action-outcome association that is necessary for successful performance in standard tests of impulsive action. This action-outcome association opposes the initially learned excitatory association in which the action led to reward. The ability to withhold responding, or inhibitory control, is often ascribed to higher executive functions and decision-making processes controlled by cortical areas, which act to modulate subcortical regions involved in “drive” (Dalley et al., 2011; Bari and Robbins, 2013). However, deficits in response inhibition also arise locally within lower neural areas involved in the volitional process (such as the NAc, which is usually associated with the “drive” component but may also have an inhibitory role). There also may be separable component processes underlying the acquisition/learning and the expression of inhibitory control, which would require carefully designed behavioral studies to separate.

### Inhibition and Classical Conditioning

Learning about inhibitory associations is an important component to consider in understanding response inhibition in impulsive action. This is distinct from the behavioral/motor expression. Deficits in inhibitory learning could be a cause of deficits in response inhibition, or alternatively, could be intact with the impulsivity arising at other levels of processing. While impulsivity itself is not defined in the context of classical conditioning, a behavioral output may appear impulsive if there are underlying deficits in inhibitory learning. For example, an impulsive behavior could result from the lack of learning of the response omission – reward association, or from a decreased ability to withhold a response. The acquisition of inhibitory learning has been studied extensively in the context of classical conditioning.

A primary area of inhibitory learning is extinction, where a new inhibitory memory is acquired to compete against a previously established excitatory memory. Importantly, extinction is not an erasure of a memory, but rather a competition of parallel associations, where old memories/behaviors can spontaneously renew (Bouton, 1993; Todd et al., 2014; Bouton et al., 2021). A deficit in the formation of the new inhibitory association or a failure of this association to successfully compete with the excitatory association, could result in altered impulsivity in classic tests of impulsive action. However, though there are many parallels between Pavlovian and operant extinction, there are also clear dissociations; for example, Pavlovian extinction does not usually transfer between conditioned stimuli, but operant extinction does (Trask et al., 2017; Bouton et al., 2021). Neurally, both the BLA and the infralimbic cortex, among others, are all involved in both Pavlovian and operant extinction, but the NAc shell is especially implicated in operant extinction (Millan and McNally, 2011; reviewed in Bouton et al., 2021). The hippocampus also seems to be involved in behavioral inhibition during extinction, as lesions to this region prevent extinction of a previously classically conditioned appetitive stimulus (Chan et al., 2003). All of these regions have also been implicated in the modulation of impulsive action, suggesting that deficits in extinction behavior may be involved in some presentations of impulsivity, or rely on dysfunction in similar neural mechanisms.

Another Pavlovian behavioral paradigm which could be useful in understanding the role of inhibitory learning in impulsive action is conditioned inhibition (reviewed by Sosa and dos Santos, 2019). Conditioned inhibition is a form of classical inhibitory learning where an inhibitory cue indicates the absence of an outcome when it is paired as a compound with a normally excitatory cue (A+, AX−; Pavlov, 1927). This inhibitor cue can then “transfer” and reduce responding when paired with other excitatory cues (BX−; Holland, 1989). Impulsive subjects which have diminished inhibitory control in operant paradigms may also fail to inhibit responding for the inhibitor-excitor compound cue in a test of Pavlovian conditioned inhibition, potentially suggesting common underlying mechanisms. Accordingly, He et al. (2011) found decreased expression of conditioned inhibition in a clinical population with personality disorders, often characterized by disinhibition and impulsivity. However, to dissociate the acquisition of this inhibitory learning from behavioral expression, acute time-limited experiments using optogenetic or chemogenetic inactivation of relevant neural targets during training vs. recall testing may be necessary.

Another version of Pavlovian inhibitory learning is negative occasion setting in which an inhibitory cue indicates that an outcome will not occur when presented in sequence with a normally excitatory cue (A+, X → A−). In this case, the conditioning is specific to the trained set of cues, and the inhibitor does not usually transfer to a different excitatory cue (Holland, 1989). Adolescent rats take longer to discriminate between reinforced and non-reinforced trials in a negative occasion setting paradigm when compared to preadolescents and adults, possibly due the functional immaturity of the PFC during this developmental period (Meyer and Bucci, 2017). Indeed, the prelimbic region of the PFC is necessary for learning this discrimination negative occasion setting, but not expressing it following training (MacLeod and Bucci, 2010). Additionally, these findings were replicated in a conditioned inhibition paradigm, where Meyer and Bucci (2014) found that lesions of the prelimbic region of the PFC decreased acquisition of conditioned inhibition learning, whereas lesions of the infralimbic cortex decreased behavioral expression following successful discrimination. Further testing inhibitory learning processes in established models for impulsive action or clinical populations are important next steps. These classical conditioning experiments could help elucidate the underlying behavioral/cognitive deficits present in specific cases of impulsivity, as well as suggesting potential shared neural substrates.

#### Contributions of Serotonin to Inhibitory Classical Conditioning

Though serotonin is strongly implicated in behavioral inhibition in instrumental conditioning, it is also involved in the acquisition and expression of inhibitory learning in these classical conditioning experiments. Lister et al. (1996) found that ablation of serotonergic pathways in rats reduced the acquisition of inhibitory associations, but left excitatory associations intact in a conditioned inhibition task. Knockout of the serotonin-transporter in rats also results in reduced latent inhibition, which is when a previously unpaired stimulus acquires inhibitory properties (Nonkes et al., 2012). Additionally, serotonin depletion impairs both Pavlovian and instrumental reversal learning, resulting in perseverative responding for a previously rewarded or safe stimulus (Cools et al., 2008; Kanen et al., 2021). This could be interpreted as a failure of behavioral flexibility, or as a failure to learn/express a new inhibitory association. One concept that unites these findings together is that serotonin signaling may play a role in the processing of aversive outcomes (including low reward, reward absence, or punishment; Crockett et al., 2012; Geurts et al., 2013; Unger et al., 2020). Specifically, serotonergic dysfunction (induced by tryptophan depletion) may cause a more positive estimation of the value of aversive outcomes, resulting in disinhibition of responding in both classical and operant conditioning (Dayan and Huys, 2008).

Serotonin is also involved in the extinction of classical conditioning. However, most research in this area has been conducted in fear extinction, rather than extinction of appetitive cues which correspond better to the inhibitory associations necessary for typical reward-based tests of impulsive action (though Pereyra et al., 2021b do describe serotonin effects on extinction of *operant* responding for reward). In fear conditioning, knockout of the serotonin transporter impairs extinction recall, though here the effect may be through the retention or expression, rather than the acquisition, of the new inhibitory association (Wellman et al., 2007; Narayanan et al., 2011; furthered reviewed in Bauer, 2015). Consideration of extending the role of serotonin signaling from operant behavioral inhibition to appetitive classical inhibitory conditioning (e.g., in tests of conditioned inhibition, negative occasion setting, and extinction) may help parse the behavioral and circuit mechanisms through which serotonin impacts inhibition. Importantly, inhibitory learning could be an important avenue through which serotonin modulates impulse control.

### Impulsive Action and Response Inhibition

Though disordered impulsivity could occur because of differences in inhibitory Pavlovian associations, it is defined in the context of operant conditioning requiring inhibition of an action to obtain reward. Nevertheless, similarly to classically conditioned response inhibition, the inhibitory “brake” seems to rely heavily on prefrontal regions upstream of subcortical reward areas (see Bari and Robbins, 2013 for an extensive review of their search). In humans, several fMRI studies have identified neural correlates of inhibitory control during tests of impulsive action. Activity in the vlPFC was associated with successful response inhibition in no-go trials for larger monetary rewards in an incentivized inhibition task (Leong et al., 2018). Additionally, using a stop signal task, Weafer et al. (2019) found that decreased activity in the right PFC during response inhibition was associated with higher left ventral striatum activity during reward receipt, suggesting negative functional association between inhibitory control and reward drive modulated through cortico-striatal connections. The anterior cingulate cortex (ACC) has also been implicated in impulse control in subjects with ADHD (Baytunca et al., 2021), and its activity is related to error processing in a Go/No-go task (Hester et al., 2004).

There is also a large literature investigating the neural circuitry underlying cortical control of response inhibition in preclinical models. Pharmacological inactivation of various regions of the mPFC, especially the prelimbic and infralimbic regions, resulted in a loss of inhibitory control on no-go trials in a response inhibition task which included shock punishments (Verharen et al., 2019). Chemogenetic activation of the vmPFC to NAc shell pathway decreases motor impulsivity in a 1CSRTT and binge-eating in rats, suggesting that these higher order areas have inhibitory control over reward processing (Anastasio et al., 2019). Indeed, using optogenetics, Li et al. (2020) found that another cortical-subcortical connection from the dmPFC to the STN in mice was important for response inhibition in a Go/No-go task. In the ACC, inhibitory G proteins are involved in the control of premature responding in the 5CSRTT (van der Veen et al., 2021). Interestingly, these studies manipulate their pathways/regions only after subjects acquired baseline training performance, suggesting that these pathways play a role in the behavioral expression of inhibition, not necessarily the learning itself. There is also convergent human and animal evidence for a role of the OFC in response inhibition (reviewed in Winstanley et al., 2010), however, single-unit recordings by Bryden and Roesch (2015) revealed that OFC neuron activity seems to support the separation of similar actions rather than inhibition independently.

Beyond the cortex, there is also evidence for the contribution of subcortical areas to response inhibition during tests of impulsive action. Deep brain stimulation of the NAc core in rats decreased impulsivity as measured by premature responding in a reaction time test, while stimulation of the NAc shell increased impulsivity, suggesting that the different subregions of the NAc may functionally support both excitation and inhibition in pursuit of reward (Sesia et al., 2008). Also, in the NAc, local inhibitory control may occur through the activity of fast-spiking interneurons, which seem to constrain impulsive action in the 5CSRTT, likely by inhibiting signaling of medium spiny neurons (Pisansky et al., 2019). Finally, dopamine signaling in the dorsal striatum is also important for response inhibition in a stop-signal task (Robertson et al., 2015). Together, all these studies suggest that the inhibitory control of impulsive action relies both on cortical and local sub-cortical control of reward processing areas.

#### Contributions of Serotonin to Inhibitory Control of Impulsivity

Overall, the serotonin system is well-positioned to impact impulsive action through its ability to modulate components of this inhibitory control system. Serotonergic neurons from the DRN innervate many cortical (and some subcortical) regions implicated in behavioral inhibition (Figure 2B), and signal through a number of different serotonin receptors (Table 1). Classic research which implicates serotonin in the regulation of anxiety behavior, is sometimes extended to the behavioral inhibition concept. For example freezing behavior in response to aversive stimuli (e.g., Wise et al., 1973) or a lack of approach in a conflict test (Graeff et al., 1997) may be viewed as inhibited behavioral responses. However, while serotonergic neurons do respond to such anxiogenic and aversive stimuli (Wise et al., 1973; Ren et al., 2018), clinical observations failed to support the theory that serotonergic signaling generated aversion. Soubrié (1986) proposed a simple resolution to this apparent conflict: serotonin encoded not anxiety but the “stop” signal that such an emotional experience occasions. Subsequently, a great deal of work has sought to characterize the precise nature of serotonin’s role in behavioral inhibition in both punishment (e.g., Crockett et al., 2009) and reward (Clark et al., 2005; Eagle et al., 2009; Nonkes and Homberg, 2013; Miyazaki et al., 2014, 2020; Nonkes et al., 2014; Odland et al., 2021).

Whereas serotonin’s contributions to reward can be assessed directly outside of tests of impulsivity (as discussed), its role in behavioral inhibition is harder to extricate since tests of impulsive action are most commonly tests of behavioral restraint. Nevertheless, both behavioral evidence and a review of areas in which serotonin acts on behavioral restraint support a role for serotonin in “brake” processes. Global 5-HT depletion increases premature responding in the 5CSRT task (Harrison et al., 1997; Winstanley et al., 2004) and impairs behavioral restraint on “no-go” trials in the Go/No-go task (Harrison et al., 1999a; Masaki et al., 2006). Critically, the deficits induced by such depletion are specific to impulse control during the action preparation phase of behavior: both the ability to cease ongoing behavior, tested with the stop-signal reaction time task (Eagle et al., 2008a), and preference for smaller, immediate rewards in delay discounting (Winstanley et al., 2003; Worbe et al., 2014) are *insensitive* to serotonergic manipulation. In the other direction, stimulating serotonergic neurons enhances the ability to wait for reward delivery in “patience” based tasks, but is not, itself, rewarding (Miyazaki et al., 2014, 2018; Fonseca et al., 2015). Further, serotonergic release differentially mediates waiting through actions in different cortical areas (Miyazaki et al., 2020). Specifically, stimulation of 5-HT release in the mPFC improves “patience” only during periods of waiting uncertainty (Miyazaki et al., 2020). Because the mPFC is generally thought to regulate the timing of behavior and encodes event and delay durations (Narayanan and Laubach, 2006; Kim et al., 2009; Xu et al., 2014; Tiganj et al., 2017), the selectivity of the effect of stimulated release suggests that prefrontal serotonin may contribute to action inhibition through a role in response timing. The many functions of the mPFC are mediated by dissociable, heterogeneous populations of projection neurons, which display a wide range of responses to affective stimuli (Grant et al., 2021) and regulate discrete aspects of motivated behavior (Otis et al., 2017, 2019). Serotonin is well positioned to modulate general cortical synchrony and the balanced activity of output pathways through tuning the activity of both inhibitory microcircuits and projection neurons (Puig and Gulledge, 2011; Dembrow and Johnston, 2014), as well as regulating the general cortical response to affective stimuli (Pereyra et al., 2021a).

The mPFC receives strong serotonin innervation from the DRN and is critical for behavioral control through both action selection and timing. Paradoxically, though elevated tonic extracellular levels of 5-HT in mPFC correlate with higher impulsive action in the 5CSRTT (Dalley et al., 2002), directly stimulating serotonin release in mPFC terminals increases wait times (i.e., decreases impulsivity) in a delayed reward task (Miyazaki et al., 2020). Resolution of this conflict will likely require characterization of both phasic serotonin release in the mPFC and neural responses to said release during such tasks. Furthermore, serotonergic neurons co-release glutamate in numerous other areas, including the amygdala (Sengupta et al., 2017) and VTA (Wang H. L. et al., 2019) while the presence of glutamate-serotonin co-transmission has not been characterized within the mPFC, DRN terminals in this area show robust coexpression of SERT and VGLUT3 (Belmer et al., 2019), indicating that the contribution of glutamatergic signaling to DRN stimulation must be considered.

The ACC is another locus for behavioral control that is modulated by serotonin. Altered serotoergic signaling in humans is associated with altered ACC activity that correlates with impoverished action monitoring and behavioral restraint (Holmes et al., 2010; Hong et al., 2018). 5-HT_1B_ receptor binding within the ACC is strongly associated with inhibiting responses to stimuli in an emotional Go/No-go task (da Cunha-Bang et al., 2017). Serotonin also acts at 5-HT_1A_ (Tian et al., 2017) and complexed 5-HT_2A/C_ receptors (Price et al., 2019) within the ACC. However, the precise role that these latter receptors play in behavior remains unclear. The behavioral consequences of 5-HT_2C_ activation or inactivation are mixed, likely due to the lack of receptor specificity in some pharmacological manipulations, with some drugs impacting both the 2C and 2A receptors. Across multiple studies, systemic 5-HT_2*C*_ agonism has been observed to increase (Koskinen et al., 2000b; Blokland et al., 2005) or decrease (Fletcher et al., 2007, 2013) premature responding in the 5CSRTT. More specific systemic antagonism of the 5-HT_2A_ receptors reduces premature responding in the 5CSRTT (Fletcher et al., 2007). Interestingly, when infused directly into the ACC, a 5-HT_2*A/2C*_ agonist (which had increased impulsive action in the 5CSRTT when administered systemically in the same study) had no effect on impulsivity (Koskinen et al., 2000b). Though the precise functional roles of ACC serotonin receptors clearly remain to be determined, they are likely related to the ACC’s established roles in both maintaining representations of desired outcomes and inhibiting behaviors that interfere with outcome acquisition (Berkman et al., 2012). These functions of the ACC are accomplished, in part, by cortical inhibition of stimulus-response associations within the striatum to permit the control of behavior by action-outcome contingencies (Cools et al., 2008), and serotonin is a known modulator of these projections.

Serotonin also acts directly in both ventral and dorsal striatum, important targets of both the mPFC and ACC in their regulation of behavior, to regulate motivation and impulsivity. While serotonin in the ventral striatum *counters* anticipatory encoding of proximal rewards, serotonin in the dorsal striatum *enables* prospective encoding of distal rewards (Tanaka et al., 2007). In the latter system, serotonin facilitates information processing through the interplay between cortical input and 5-HT_2A_/5-HT_2C_ signaling (Agnoli and Carli, 2012). In the ventral striatum both 5-HT_2A_ and 5-HT_2C_ antagonism reduce impulsivity, and 5-HT_2A_ antagonism appears to do this by generally suppressing motivated responding (Robinson et al., 2008). Serotonin depletion in the NAc increases the rate of responding in the DRL without appearing to influence motor impulsivity and is theorized to reflect a decreased tolerance for delayed reward (Fletcher et al., 2009). However, directly stimulating serotonergic terminals in the NAc does not increase waiting in a patience-based task (Miyazaki et al., 2020). Thus, serotonergic signaling within the ventral striatum appears to mediate proximal reward response and approach drive, while within the dorsal striatum it facilitates control of behavior in the face of delayed rewards.

In summary, the literature supports the following conclusions: (1) Serotonin receptors are positioned to regulate cortical microcircuits and projection neurons. (2) Serotonergic manipulations within prefrontal cortices and striatal outputs alter impulsive behavior. (3) The described functions of these cortical regions align best with “brake” processes in behavioral restraint rather than “drive” processes. To build a model of serotonergic impulsivity-regulation within cortical circuits, these ideas would ideally be integrated in a cohesive framework. For example, future work could characterize the role and impact of serotonergic signaling on the activity of functionally discrete cortical subpopulations (e.g., Hart et al., 2018; Grant et al., 2021). Causally linking serotonergic signaling within corticostriatal circuits to behavioral inhibition would be the next step. Behavioral assays targeted at cortical control processes including those targeting timing or action monitoring may help identify specific “brake” functions altered in impulsive animals. Meanwhile, tests sensitive to altered reward and motivation may serve to *exclude* changes in “drive” processes.

## Discussion: Impulsivity as an Imbalance of Systems and Future Directions

Through this review, we have provided an overview of the behavioral and neural systems underlying impulsive action. Dysregulations of either reward or inhibition can create an imbalance of the neural systems responsible for impulse control. Neurally, we suggest that widespread DRN serotonergic projections (Figure 2) place serotonin, signaling through its various receptor types (Table 1), in a prime position to modulate both the excitatory and inhibitory components of these systems. Indeed, there may be multiple serotonin subsystems which separably mediate responses to rewarding or aversive outcomes (Ren et al., 2018). Either excess excitation or decreased inhibitory control could result in increased impulsive action as observed by a decreased ability to stop or withhold responding. In this case, the initially learned “go” association overrides the “no-go” or stop association. Increased impulsivity could also be the result of altered activity in both drive and brake processes. Ultimately, both processes compete over controlling the same endpoint: motor output. For animals to achieve efficient, flexible behavior, the drive and brake circuitry must each be responsive to task demands in guiding action selection.

Adolescence is an interesting case which allows us to probe the role of these two processes and how serotonin influences the balance. Specifically, adolescence is a developmental period characterized by increased impulsivity, risky decision making, and hyper reward-sensitivity. In the dimension of impulsive action, compared to children and adults, teenagers have more false alarms for no-go cues in the Go/No-go test (Somerville et al., 2011; Dreyfuss et al., 2014). This heightened impulsive action is thought to be the result of the linear development of the PFC and the nonlinear development of the ventral striatum and other components of the reward system, which peak in sensitivity during adolescence (Blakemore and Robbins, 2012). This results in an imbalance between the subcortical systems which motivate behavior and the cortical systems providing inhibitory control compared to childhood and adulthood (Casey et al., 2011). Substance use disorders have also been considered through a similar lens, with both increased appetitive drive and disordered executive control potentially resulting in impulsive behavior, though the extent to which impulsivity is causal or resultant to addiction is unclear (Bechara, 2005; Camchong et al., 2014; Jentsch et al., 2014; Kozak et al., 2019).

**FIGURE 3:**
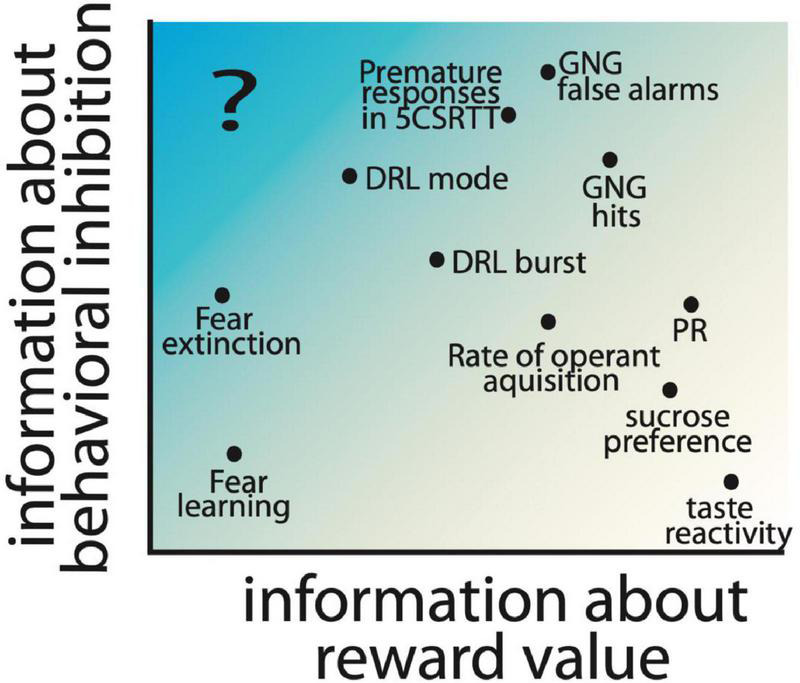
Hypothesized contribution of reward value and behavioral inhibition factors to commonly used preclinical behavioral assays is shown based on the location of measures on the reward and inhibition axes. Parameters extracted from commonly used assays of impulsive action, including Go/No-go (GNG), 5 choice serial reaction time task (5CSRTT), and differential reinforcement of low-rate responding (DRL) are included, as well as other operant tasks which measure motivation, e.g., progressive ratio (PR) and hedonic responses (sucrose preference and taste reactivity). Fear learning and rate of extinction of fear learning are also included as measures with low information about reward value. Highlighted with a “?” is an information space that would provide measures of impulsivity independent of reward, and has limited preclinical behavioral assays.

Importantly, the imbalance of reward and inhibitory processing could be the result of dysfunction of many different regions, cell types, and/or receptor types, which may each result in an impulsive action phenotype, albeit through different neural and behavioral processes. Therefore, careful dissections of the processes which contribute to impulsive action allows for the fractionation of different paths to an overall impulsive phenotype. Testing may use non-traditional tests for the study of impulsivity, including the consideration of Pavlovian and instrumental learning processes, the expression of behavioral inhibition, and reward processes. For example, in Figure 3, we show a chart with reward and behavioral inhibition as hypothetical dimensions characterizing different behavioral measures. Notably, tests like taste reactivity primarily measure a reward-related behavior; on the other hand tests like the Go/No-go are considered measures of impulsive action, but are also influenced by reward value (Desrochers et al., 2021). Desrochers et al. found that increasing reward quantity increases false alarm rates for no-go trials in control mice, while decreasing reward quantity reduced this measure in normally impulsive mice lacking the 5-HT_1B_ receptor. This suggests that some traditional measures of impulsive action are intrinsically tied to reward related behaviors. However, we highlight the idea that additional approaches to measuring impulsivity in the absence of learned appetitive motivators may allow the dissociation of reward processes from behavioral inhibition (see Figure 3). It is unclear to us whether any existing tests could specifically be used to measure impulsive action in the absence of reward. A possibility may be active avoidance, where animals learn to avoid the side of a shuttle box associated with an aversive outcome. Subjects with decreased behavioral inhibition may have enhanced active avoidance behaviors (more rapid acquisition of the behavior) in this task; indeed, selectively bred Roman-high avoidance rats also have increased premature responding in the 5CSRTT compared to low-avoidance rats (Moreno et al., 2010). Additionally, in these rat strains, 5-HT_2A_ binding levels are higher in the high-avoidance rats and correlate with impulsivity in the 5CSRTT (Klein et al., 2014). This suggests a role for serotonin signaling in behavioral inhibition, independent of reward, which could be further studied using an active avoidance test. More studies testing traditional models for pathological impulsivity in active avoidance paradigms would be helpful to understand if this would be a useful approach to measuring impulsive action without an appetitive conditioning paradigm.

In conclusion, we support consideration of components of impulsivity beyond the overarching subtypes (i.e., action vs. choice), to include specific cognitive and behavioral substrates. Studies of disordered impulse control, both in human and animal models, would ideally use multiple and varied behavioral tests to determine the underlying component processes and tease apart influences of reward from inhibitory control. The understanding of the contributions of these processes can provide tractable targets for pursuing neural circuit mechanisms and potentially more individualized treatment approaches for pathological impulsivity. In particular, we propose that serotonin signaling is an important mechanism to explore in this context to understand the behavioral and neural bases of the control of impulsive action.

## Author Contributions

SD, MS, and KN contributed to the ideas, wrote the manuscript, and approved the final version.

## Conflict of Interest

The authors declare that the research was conducted in the absence of any commercial or financial relationships that could be construed as a potential conflict of interest.

## Publisher’s Note

All claims expressed in this article are solely those of the authors and do not necessarily represent those of their affiliated organizations, or those of the publisher, the editors and the reviewers. Any product that may be evaluated in this article, or claim that may be made by its manufacturer, is not guaranteed or endorsed by the publisher.
